# Community-level consumption of antibiotics according to the AWaRe (Access, Watch, Reserve) classification in rural Vietnam

**DOI:** 10.1093/jacamr/dlaa048

**Published:** 2020-09-14

**Authors:** Nam Vinh Nguyen, Nga Thi Thuy Do, Chuc Thi Kim Nguyen, Toan Khanh Tran, Phuc Dang Ho, Hanh Hong Nguyen, Huong Thi Lan Vu, Heiman F L Wertheim, H Rogier van Doorn, Sonia Lewycka

**Affiliations:** 1 Oxford University Clinical Research Unit, Hanoi Unit, Hanoi, Vietnam; 2 Hanoi University of Pharmacy, Hanoi, Vietnam; 3 Family Medicine Department, Hanoi Medical University, Hanoi, Vietnam; 4 FilaBavi Health Demographic and Surveillance Site, Hanoi, Vietnam; 5 National Institute of Mathematics, Hanoi, Vietnam; 6 Department of Medical Microbiology and RadboudUMC Center for Infectious Diseases, RadboudUMC, Nijmegen, Netherlands; 7 University of Oxford Centre for Tropical Medicine and Global Health, Nuffield Department of Medicine, Oxford, UK

## Abstract

**Objectives:**

To review community-level consumption of antibiotics in rural Vietnam, according to the WHO Access, Watch, Reserve (AWaRe) classification of 2019, and identify factors associated with the choice of these antibiotics.

**Methods:**

In this cross-sectional study, data on antibiotic purchases were collected through a customer exit survey of 20 community antibiotic suppliers in Ba Vi District, Hanoi, between September 2017 and July 2018. Antibiotic consumption was estimated through the number of antibiotic encounters, the number of DDDs supplied and the number of treatment days (DOTs) with antibiotics, and analysed according to the AWaRe classification. The factors associated with watch-group antibiotic supply were identified through multivariable logistic regression analysis.

**Results:**

In total, there were 1342 antibiotic encounters, with access-group antibiotics supplied in 792 encounters (59.0%), watch-group antibiotics supplied in 527 encounters (39.3%) and not-recommended antibiotics supplied in 23 encounters (1.7%). No reserve-group antibiotics were supplied. In children, the consumption of watch-group antibiotics dominated in all three measures (54.8% of encounters, 53.0% of DOTs and 53.6% of DDDs). Factors associated with a higher likelihood of watch-group antibiotic supply were: private pharmacy (OR, 4.23; 95% CI, 2.8–6.38; *P *<* *0.001), non-prescription antibiotic sale (OR, 2.62; 95% CI, 1.78–3.87; *P *<* *0.001) and children (OR, 2.56; 95% CI, 1.84–3.55; *P *<* *0.001).

**Conclusions:**

High consumption of watch-group antibiotics was observed, especially for use in children. The frequent supply of watch-group antibiotics at private pharmacies reconfirms the need for implementing pharmacy-targeted interventions in Vietnam.

## Introduction

Overuse of antibiotics is a major public health issue.^1^^,^^2^ In the current era of increasing antibiotic resistance and scarcity of new antibiotic development, enhancing rational use of antibiotics is included as one of five strategic objectives of the WHO global action plan on antibiotic resistance, as well as the Vietnamese National Action Plan.^3–5^ However, despite international efforts to regulate antibiotic use, the global consumption of antibiotics has continued to increase over time.^1^^,^^6–8^ A recent study shows that global antibiotic consumption increased by 65% between 2000 and 2015, primarily driven by low- and middle-income countries (LMICs) that are experiencing economic growth. The total amount of antibiotics consumed in LMICs, which was at a similar level to that in high-income countries (HICs) in 2000, reached nearly 2.5 times that in HICs by 2015.^9^

In Vietnam, economic reforms during the 1980s and 1990s helped transform the country from a low-income to a lower middle-income country by 2013. This reform also led to an increased number of private pharmacies and drug sellers, and unregulated access to antibiotics.^10^^,^^11^ Although laws exist to restrict antibiotic use, the enforcement of these has remained insufficient. In addition, most antibiotic stewardship programmes have only focused on hospitals, where resistant infections are identified, while the vast majority of the antibiotics are consumed in the community.^12^^,^^13^ These antibiotics are either prescribed by healthcare professionals or purchased directly by consumers without prescription.^14^ In Vietnam, antibiotic self-medication through private pharmacies is very common. According to a previous study, 88% of antibiotics dispensed in urban pharmacies and 91% in rural pharmacies were without prescription. This practice was driven by poor knowledge among pharmacy staff and customers on antibiotics and resistance, as well as pharmacists’ fear of losing customers, especially in rural areas.^15^

At the 21st meeting of the WHO Expert Committee on the Selection and Use of Essential Medicines in 2017 the WHO Model List of Essential Medicines (EML) and the Model List of Essential Medicines for Children (EMLc) were reviewed and updated.^16^^,^^17^ An important addition was a new categorization of antibacterials into three groups, which emphasizes empiric treatment choices for common, community-acquired infections that are broadly applicable in the majority of countries. The WHO recommended use of this classification to assist in the development of antibiotic stewardship at local, national and global levels. In October 2019, this classification was updated and reformed as a classification database. Accordingly, 180 common antibacterials are classified into three groups: access, watch or reserve (AWaRe). Access-group antibiotics include 49 antibiotics that have activity against a wide range of commonly encountered susceptible pathogens and a lower resistance potential than antibiotics in the other groups. Watch-group antibiotics include 110 antibiotics that have higher resistance potential. Finally, 22 reserve-group antibiotics should be considered antibiotics of last resort, which should be tailored to highly specific patients and settings, when all alternatives have failed or are not suitable. The database also lists those antibiotics whose use is not recommended by WHO, namely fixed-dose combinations of multiple broad-spectrum antibiotics that lack evidence-based indications for use or recommendations in high-quality international guidelines.

This classification has been used in several antibiotic-use studies to review the current levels of the use of watch- and reserve-group antibiotics in different settings.^18^^,^^19^ However, a limitation of these studies is the inclusion of hospital antibiotic consumption data, which also included hospital-acquired infections, while the WHO EML primarily focuses on antibacterial choices for common, community-acquired infections. Furthermore, the use of integrated antibiotic sales data means that individual-level factors associated with the use of different groups of antibiotics could not be explored. Lastly, as the classification was changed in October 2019, after publication of these data, they still use the old classification.

Therefore, we conducted this study to assess community-level antibiotic consumption according to the WHO AWaRe groups and to identify the factors associated with the choice of watch- and reserve-group antibiotics in a rural area in Vietnam, an LMIC in Southeast Asia.

## Methods

### Study design and setting

This cross-sectional study was part of the ABACUS (AntiBiotic Access and Use) study.^20^ The primary objective of ABACUS was to identify targets for community-based, tailored intervention strategies to promote appropriate antibiotic use across communities in six LMICs in Asia (Bangladesh, Thailand and Vietnam) and Africa (Mozambique, Ghana and South Africa). For each study site, including Vietnam, all possible antibiotic dispensing points (antibiotic suppliers) were mapped and were eligible to participate in this study. This included any formal or informal antibiotic supplier in the community, from public hospital pharmacy to street vendor.

Our study used data collected through face-to-face customer exit surveys at 20 antibiotic suppliers, between September 2017 and July 2018, in Ba Vi District, located 60 km west of the centre of Hanoi, Vietnam. It covers 410 km^2^ and has a population of 274 000 people within 31 communes. The healthcare system includes a district hospital with 300 beds, 3 public polyclinics and 31 commune health stations and village health workers, as well as private facilities and pharmacies.^21^ Private pharmacies have been shown to be the most accessible and accessed drug-selling points in this area.^15^

### Study participants

In Ba Vi District, 20 of 502 antibiotic suppliers were selected for customer exit interviews. The selection was based on highest rank in number of daily antibiotic encounters. Four suppliers refused to participate in the survey at our first invitation and were replaced by four other suppliers. The final selected suppliers included 5 drug outlets in public health settings (1 from a district hospital, 1 from a local polyclinic and 3 from commune health stations) and 15 private pharmacies. To limit biases caused by seasonal variations, the survey was performed four times over a 1 year period between September 2017 and July 2018. We selected 30 encounters per supplier from 20 suppliers to represent the study population, based on WHO and International Network for Rational Use of Drugs (WHO/INRUD) methodology.^22^

At the selected suppliers, each customer who left the pharmacy during one of the four 3 day data collection phases was approached and asked whether antibiotics had been supplied to them. In the case of an antibiotic encounter, the customer was asked to participate in the customer exit interview. To be included in the study, customers had to be willing to disclose whether antibiotics were supplied to them and consent to participate in an interview. Recruitment of participants was stopped when either the end of that survey round was reached or 30 customers with antibiotic encounters were recruited.

### Data collection and management

Demographics of antibiotic purchasers, antibiotic supply (name, dosage, number of units supplied and length of treatment) and self-reported health conditions for requesting antibiotics (health problems or symptoms) were collected from antibiotic purchasers through face-to-face interviews using a structured questionnaire (Appendix S1, available as Supplementary data at *JAC-AMR* Online). Antibiotic purchases for children were defined according to the respondent’s report, with no pre-specified age range. All of the interviews were conducted by field workers of the FilaBavi Health Demographic and Surveillance Site, who had previous experience in data collection and were trained on the content of the questionnaire as well as interview skills. The REDCap data management platform (redcap.core.wits.ac.za) was used for data collection.

### Data analysis

We presented antibiotic consumption in three measures: antibiotic encounters, DDDs and days of treatment (DOTs). For each antibiotic encounter, the number of DDDs was measured through the number of standard units supplied, the strength of antibiotic products and the DDD assigned for each drug by the WHO Collaborating Centre and the WHO International Working Group on Drug Statistics Methodology. In this study, we applied the DDD for both adults and children since the DDD database for children has not been created. For DOT measurement, the number of DOTs was defined according to the respondent’s report.

The patterns of use were described according to the 2019 WHO AWaRe antibiotic classification, in which antibiotics were classified into four groups: access, watch, reserve and not recommended.^16^ For encounters with a combination of antibiotics, the classification of the encounter would be based on the higher restricted antibiotic. For instance, if the customer’s basket contained both watch-group and access-group antibiotics, the encounter would be classified as a watch-group antibiotic encounter. We also used the WHO Anatomical Therapeutic Chemical (WHO ATC) classification to rank the consumption of pharmacological classes of antibiotics.

We performed multivariable logistic regression analysis to review the factors associated with the choice of watch- and reserve-group antibiotics because both of them are recommended by WHO as key targets of antibiotic stewardship programmes and monitoring.^16^^,^^17^ Since the use of reserve-group antibiotics was not observed in our study population (see the Results section), our analysis only reviewed the factors associated with the choice of watch-group antibiotics.

R version 3.5.1 was used for all data analyses.

### Ethics

The study protocol was reviewed by the Oxford University Tropical Research Ethics Committee (OxTREC, Reference: 31-15) and the National Vietnamese ethical committees [Vietnam Ministry of Health (MoH) Institutional Review Board (IRB) (6670/QD-BYT)] and the Ethical Committee of Hanoi Medical University (No. 195/HMU IRB).

## Results

A total of 1404 antibiotic encounters were included in this study, of which 1342 antibiotic encounters were related to human use. Customers’ characteristics from these 1342 antibiotic encounters are presented in Table 1. Customers’ median age was 40 years (IQR: 30–55) and 69.0% were female. In 389 encounters (28.9%), antibiotics were purchased for children. Antibiotics were purchased without prescription in 773 encounters (57.6%). Cough (724 cases, 53.9%), sore throat (644 cases, 48.0%), fever (447 cases, 33.3%) and runny nose (378 cases, 28.2%) were the most common health problems and symptoms for purchasing antibiotics.

**Table 1.** Characteristics of antibiotic customers

	Number of antibiotic encounters (*n *=* *1342)	
	*n* ^a^	%^a^
Antibiotic customer characteristics		
age, median (IQR) (years)	40	(30–55)
female gender	926	69.0
antibiotic purchase for children	389	28.9
non-prescription sale of antibiotics	773	57.6
private pharmacies	863	64.3
Reason for purchasing antibiotics		
chest pain	66	4.9
cough	724	53.9
dental symptoms	37	2.8
dyspnoea	71	5.3
ear and eye symptoms	36	2.7
fever	447	33.3
gastrointestinal symptoms	125	9.3
gynaecological symptoms	23	1.7
headache	162	12.1
musculoskeletal symptoms	16	1.2
runny nose	378	28.2
skin and soft tissue symptoms	28	2.1
surgery-related symptoms	4	0.3
sore throat	644	48.0
urinary tract symptoms	36	2.7
wound	57	4.2
others	51	3.8

a Unless otherwise stated.

Table 2 shows consumption levels of access-, watch- and reserve-group antibiotics, estimated by the number of antibiotic encounters, the number of DOTs and the number of DDDs supplied. In the total of 1342 antibiotic encounters, access-group antibiotics were provided in 792 encounters (59.0%), watch-group antibiotics in 527 encounters (39.3%) and not-recommended antibiotics in 23 encounters (1.7%). The total DOTs with antibiotics was 5889, of which 3484 (59.2%) were access-group, 2293 (38.9%) were watch-group and 112 (1.9%) were not-recommended antibiotics. The total DDDs dispensed was 7221.7 DDDs, of which 3663.8 (50.7%) were access-group, 3414.1 (47.3%) were watch-group and 143.8 (2.0%) were not-recommended antibiotics. There were no reserve group antibiotics dispensed among the interviewees. In children, the consumption of watch-group antibiotics was the highest according to all three measures: number of encounters (54.8%), number of DOTs (53.0%) and number of DDDs (53.6%).

**Table 2.** Antibiotic consumption according to WHO AWaRe antibiotic groups, estimated by antibiotic encounters, DDDs and DOTs

	Total		Adults		Children	
	*n*	%	*n*	%	*n*	%
Antibiotic encounters						
access-group antibiotics	792	59.0	625	65.6	167	42.9
watch-group antibiotics	527	39.3	314	32.9	213	54.8
reserve-group antibiotics	0	0	0	0	0	0
not-recommended antibiotics	23	1.7	14	1.5	9	2.3
total	1342	100.0	953	100.0	389	100.0
DOTs						
access-group antibiotics	3484	59.2	2722.5	65.3	761.5	44.3
watch-group antibiotics	2293	38.9	1383	33.2	910	53.0
reserve-group antibiotics	0	0	0	0	0	0
not-recommended antibiotics	112	1.9	65	1.6	47	2.7
total	5889	100.0	4170.5	100.0	1718.5	100.0
DDDs						
access-group antibiotics	3663.8	50.7	2927.5	52.9	736.3	43.6
watch-group antibiotics	3414.1	47.3	2508.9	45.3	905.2	53.6
reserve-group antibiotics	0	0	0	0	0	0
not-recommended antibiotics	143.8	2.0	97.1	1.8	46.7	2.8
total	7221.7	100.0	5533.5	100.0	1688.2	100.0

In addition to comparing antibiotic consumption by WHO AWaRE antibiotic groups, we performed an analysis of the consumption of different chemical classes of antibiotics (according to the 4th level WHO ATC classification) (Table 3). The five most consumed antibiotic groups accounted for more than 80% of total consumption. These included two from the access group: penicillins with extended spectrum [380 encounters (28.3%), 1865.8 DDDs (25.8%) and 1531.5 DOTs (26.0%)] and first-generation cephalosporins [305 encounters (22.7%), 1282.8 DDDs (17.8%) and 1349 DOTs (22.9%)]; and three from the watch group: third-generation cephalosporins [161 encounters (12%), 852.6 DDDs (11.8%) and 745 DOTs (12.6%)], macrolides [159 encounters (11.8%), 944.5 DDDs (13.1%) and 700 DOTs (11.9%)]; and second-generation cephalosporins [135 encounters (10.0%), 1147 DDDs (15.9%) and 626.5 DOTs (10.6%)].

**Table 3.** The proportional consumption (% of total consumption) of the WHO ATC chemical classes of antibiotics

WHO ATC group	Antibiotic groups	WHO AWaRe group	Estimated by					
			antibiotic encounters		number of DOTs		number of DDDs	
			*n*	%	*n*	%	*n*	%
J01CA	penicillins with extended spectrum	access	380	28.3	1531.5	26.0	1865.8	25.8
J01DB	first-generation cephalosporins	access	305	22.7	1349.5	22.9	1282.8	17.8
J01DD	third-generation cephalosporins	watch	161	12.0	745	12.6	852.6	11.8
J01EE	macrolides	watch	159	11.8	700	11.9	944.5	13.1
J01DC	second-generation cephalosporins	watch	135	10.0	626.5	10.6	1147	15.9
J01CR	combinations of penicillins, including β-lactamase inhibitors	access/not recommended	58	4.3	269.5	4.6	295.1	4.1
J01MA	fluoroquinolones	watch	37	2.8	183	3.1	322	4.5
J01EE	combinations of sulphonamides and trimethoprim, including derivatives	access	35	2.6	164.5	2.8	191.3	2.6
J01FF	lincosamides	access	34	2.5	166	2.8	162.3	2.2
J01AA	tetracyclines	access	14	1.0	51.5	0.9	93.5	1.3
J01BA	amphenicols	access	19	1.4	80	1.4	25.1	0.3
J01CE	β-lactamase-sensitive penicillins	access	5	0.4	19.5	0.3	10.4	0.1
J01XD	imidazole derivatives	access	1	0.1	10	0.2	28.3	0.4
J01GB	other aminoglycosides (aminoglycosides other than streptomycin)	access	1	0.1	1	0.0	1	0.0

**Figure 1.**
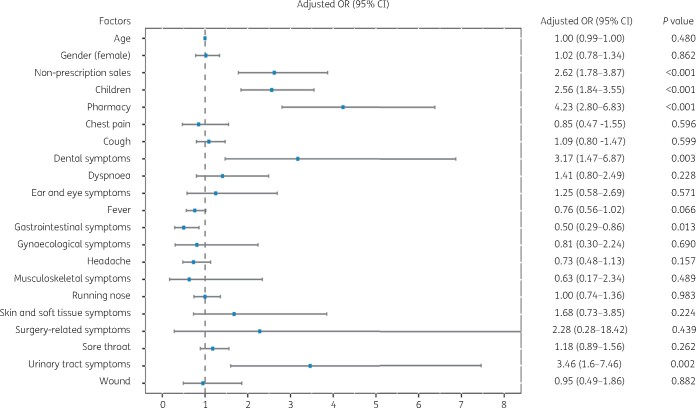
Results from multiple logistic regression analysis modelling the associations between watch-group antibiotic supply and baseline covariates.

Since the use of reserve-group antibiotics was not observed in our study population, the multivariate analysis focused on the factors associated with watch-group antibiotic supply. Our analysis shows that non-prescription sales of antibiotics (OR, 2.62; 95% CI, 1.78–3.87; *P *<* *0.001), antibiotic purchase for children (OR, 2.56; 95% CI, 1.84–3.55, *P *<* *0.001), private pharmacies (OR, 4.23; 95% CI, 2.8–6.83; *P *<* *0.001), dental symptoms (OR, 3.17; 95% CI, 1.47–6.87; *P *=* *0.003) and urinary tract symptoms (OR, 3.46; 95% CI, 1.6–7.46; *P *<* *0.001) were associated with a more frequent supply of watch-group antibiotics (Figure 1). For the populations carrying these factors, the use of different watch-group antibiotics was described in Table S1.

## Discussion

Our study shows that access-group antibiotics such as extended-spectrum penicillins (e.g. amoxicillin and ampicillin) and first-generation cephalosporins (e.g. cefalexin) were the most frequently purchased in a rural community in Vietnam, comprising nearly 60% of total antibiotic use by all three measures. This is similar to what was observed in a previous study, in which the three most commonly sold antibiotics in rural pharmacies in Vietnam were amoxicillin (27%), cefalexin (20%) and ampicillin (12%).^15^ Although these agents are recommended by WHO as first-line treatments for common community-acquired infections, it should be noted that antibiotics should be provided only if there is high suspicion of bacterial involvement.^16^^,^^23^ In this study, the majority of antibiotics were supplied without prescription to patients with respiratory tract symptoms, which have been proven to be viral in the majority of cases. In addition, for a large proportion of bacterial respiratory tract infections such as bronchitis, otitis media, sinusitis and sore throat, antibiotic treatment is not recommended.^24^

Around 40% of customers in our study population were treated with watch-group antibiotics, including oral second- and third-generation cephalosporins (cefuroxime, cefixime, cefdinir and cefpodoxime), macrolides (azithromycin, clarithromycin and erythromycin) and fluoroquinolones (levofloxacin and ciprofloxacin). Because of higher resistance potential, the use of watch-group antibiotics is only recommended as first- or second-choice treatments for a limited number of indications.^16^ The overuse of oral cephalosporins, especially cefuroxime and cefixime, can disrupt the intestinal microbiome, select for resistant organisms and is associated with a significant increased risk of *Clostridioides difficile* infection.^25^^,^^26^ For macrolides, the increased use of these agents in recent years has been shown to be linked to increased macrolide resistance in *Streptococcus pneumoniae*, especially in Asian countries.^27^^,^^28^ In Taiwan, among 276 isolates of *S. pneumoniae* collected from five major teaching hospitals, the rate of macrolide-resistant *S. pneumoniae* was over 90%.^29^ Fluoroquinolones are highly effective antibiotics for respiratory and urinary tract infections. However, the susceptibility of the pathogens causing these infections to fluoroquinolones has rapidly dropped in Asia-Pacific regions, especially in antimicrobial resistance hotspots such as Vietnam or China.^30–32^ Our subgroup analyses also increase concerns regarding inappropriate use of antibiotics in the community (Table S1). In this study, 50% of dental symptoms were treated with macrolides (mainly spiramycin/metronidazole) while amoxicillin and amoxicillin/clavulanic acid are recommended as first-line treatments in oral and dental infections.^33^ Similarly, nearly 60% of urinary tract symptoms were treated with fluoroquinolones while first-line treatments recommended for community-acquired non-complicated urinary tract infections are narrow-spectrum antibiotics such as trimethoprim and nitrofurantoin.^14^ The observation that no reserved-group antibiotic encounters were observed in this study could be explained by the fact that all of these agents are parenteral drugs, which are rarely available in rural pharmacies. However, reporting this information is important to provide a reference for similar studies in urban pharmacies in the future. In these areas, community pharmacies near big hospitals can sell parenteral antibiotics for patients whose indications are not reimbursed by the National Health Reimbursement Agency.

Another important finding of this study is the significantly higher proportion of use of watch-group antibiotics for children (nearly 55% in all three measures), confirmed to increase the likelihood of watch-group antibiotic supply in our multivariate analysis. This proportion is higher than observed in a study by Hsia *et al.*^18^ (43.1%). In this study, antibiotic sales data for children from 70 countries were ranked and Vietnam ranked fifth for consumption of watch-group antibiotics. Excessive consumption of antibiotics in children has been seen in many countries, including HICs. In South Korea, children younger than 10 years were the population most frequently treated with antibiotics.^34^ In the USA, outpatient and community-level use of broad-spectrum antibiotics in children was observed to steadily increase over time.^35^^,^^36^ There are several hypotheses for the overconsumption of watch-group antibiotics in our child population. Firstly, because of poor understanding of antibiotic use and resistance, parents often ask for newer and more expensive antibiotics, which are commonly broad-spectrum antibiotics, to treat their children. Secondly, because parents often visit pharmacies, explain their child’s symptoms and ask for medications without bringing the children with them, pharmacists might tend to choose broad-spectrum antibiotics to cover most bacterial infections. Thirdly, selling broad-spectrum antibiotics is often more profitable for antibiotic suppliers than selling narrow-spectrum antibiotics. Broad-spectrum antibiotics (e.g. azithromycin or cefixime) are often more expensive and less common compared with narrow-spectrum antibiotics (e.g. ampicillin). It is easier for suppliers to set higher retail prices for these, since parents have fewer sources for price reference and are always willing to pay higher costs for their children. Although these hypotheses need to be confirmed in future studies, restricting the supply of broad-spectrum antibiotics to children is crucial. In fact, the use of broad-spectrum antibiotics to treat common bacterial infections in children was shown to have no more clinical benefit and cause more adverse effects than narrow-spectrum antibiotics.^37^ More importantly, using these antibiotics in the early stages of life was proven to be among the key drivers of antimicrobial resistance and therefore could lead to poor efficacy of antibiotics in the future.^38^

Although antibiotic supply without valid prescription is illegal in Vietnam, more than 90% of antibiotic dispensations in private pharmacies in rural areas were without prescription.^15^ In this study, private pharmacies also had a higher proportion of dispensing watch-group antibiotics. This could be explained by differences in business models between private pharmacies and other community antibiotic suppliers (drug outlets inside public hospitals, polyclinics or commune health centres). These latter suppliers mainly provide prescribed antibiotics under the national health insurance programme, while private pharmacies exist by gaining profits from selling medications to cash customers.^10^ Therefore, the pursuit of profit might incentivize community pharmacists to sell more expensive antibiotics to customers, with less regard for appropriateness.^10^^,^^15^^,^^21^^,^^32^^,^^39^ In a previous survey with pharmacists in rural Vietnam, nearly 80% of respondents confirmed that perceived customer pressure and fear of losing customers leads to irrational antibiotic dispensation.^15^ This is similar to what was observed in India, Ethiopia and Tanzania, where refusal to dispense antibiotics was seen to possibly affect the sales of pharmacies.^14^ To tackle this issue, the law prohibiting over-the-counter sales of antibiotics needs to be more effectively enforced. In addition, other pharmacy-targeted interventions to enhance rational access and use of antibiotics should be developed. In the UK, the use of point-of-care tests in pharmacies to guide treatment decisions was shown to reduce unnecessary antibiotic use in a rural community.^40^ However, there is still a lack of evidence on the feasibility and applicability of these tests in community pharmacies in LMICs.

This study has several strengths. First, to our knowledge, this is among the first studies applying the 2019 WHO AWaRe classification in an analysis of antibiotic consumption in an LMIC. Compared with the 2017 classification, this updated version expanded the classification to cover non-essential antibiotics. Therefore, this minimizes the frequency of unclassified antibiotics in our study, which was commonly seen in previous studies.^18^^,^^19^ Second, in this study, antibiotic consumption was measured by different metrics (either the number of antibiotic encounters, the number of DDDs or the number of DOTs) to see how our study conclusions varied when the estimation method was changed. Third, the combined data on antibiotic consumption with patient-level factors allowed an exploration of risk factors for inappropriate antibiotic use. Fourth, because the data collection was conducted immediately after sales, the impact of recall bias on information about antibiotic type and dose was minimized. Finally, by using experienced interviewers who work in a demographic surveillance site, the quality of the data collected was ensured.

This study is limited by the fact that it was conducted in only the 20 largest antibiotic suppliers in one rural district. Therefore, our results may not be representative of smaller suppliers, urban communities or other parts of Vietnam. In addition, the frequency of watch-group antibiotics dispensed by large suppliers may be higher because their customers may have more severe health issues than customers of small suppliers. Our small sample size (1342), compared with the expected sample size (2400), also affects the generalizability of this study (see Table S2 for accomplishment rate of recruitment in each supplier). The fact that four suppliers refused to participate in the survey at our first invitation and were replaced by other suppliers might be related to their antibiotic supply practices and also affect generalizability. Finally, each estimation measure used in this study only gives a rough estimate of antibiotic consumption. The accuracy of DOT data could be affected by recall bias and using adult DDDs to estimate consumption in children could lead to underestimation of actual consumption in children. Therefore, we presented consumption data by both number of encounters, DOTs and DDDs to see whether there was good agreement between different estimates. This approach was used in previous studies.^41^^,^^42^

In conclusion, although access-group antibiotics were the most commonly used antibiotics in community pharmacies in a rural area in Vietnam, the consumption level of watch-group antibiotics is remarkably high, especially among children. The factors significantly associated with a higher chance of supplying watch-group antibiotics were private pharmacy, non-prescription antibiotic supply and antibiotic sales for children. These findings elaborate earlier reports and confirm existing concerns regarding inappropriate antibiotic dispensing in rural Vietnam, which may be similar in other LMICs. There is an urgent need for pharmacy-targeted interventions to tackle this problem.

## Supplementary Material

dlaa048_Supplementary_Data
