# Supplementary material for: Community-level consumption of antibiotics according to the AWaRe (Access, Watch, Reserve) classification in rural Vietnam
Source: JAC Antimicrob Resist. 2020 Sep 14;2(3):dlaa048. doi: 10.1093/jacamr/dlaa048 (PMC7497401; doi:10.1093/jacamr/dlaa048)
Supplement: dlaa048_Supplementary_Data [file dlaa048_supplementary_data.zip › Supplementary_data.docx]

**Supplementary data**

**Appendix S1. Antibiotic encounter customer exit questionnaire**

i. The following questions will be answered for each type of antibiotic supplied to a particular customer.

ii. Tablets, capsules, paediatric formulations, as well as intravenous and intramuscular antibiotics are to be assessed, but not other formulae like droplets or creams.

iii. The reason-for-encounter symptoms are based on the International Classification of Primary Care (ICPC-2e v5 May 2015).

iv. As a matter of courtesy, the antibiotic supplier/dispenser should be informed that exit interviews will be conducted outside their facility.

**CUSTOMER EXIT INTERVIEW PARTICIPANT** …….…_........._.........

[study site]_[antibiotic supplier]_[customer exit interview participant]

*Demographics*

1. Age ………………………………years
2. Sex male / female
3. Only if you are comfortable with it, I would like to ask for your permission to examine your antibiotic/s together with you. If you are not comfortable with this, I will respect this and complete the interview without inspection of your antibiotics.

*Permission provided?* yes / no

*Antibiotics*

1. What is the name of the antibiotic/s you receive (Generic name, not brand name)? ……………………………………….
2. What is the size of one tablet? …………………mg / unknown
3. How many tablets are supplied? ……………………………………….
4. For how many days is this antibiotic supplied? ………………days / unknown
5. Were these antibiotics prescribed by a health professional? Yes / no
6. For who is this antibiotic?

(tick one; Myself/ Child family member / adult family member / friend or relative / animal / unspecified / other: …../ unknown)

1. Are you comfortable with telling us about the illness for which you got this antibiotic?

Yes / no

*If no, skip to Question 12*

1. *If yes,* for what illness did you receive this antibiotic? Sore throat / cough / Flu / headache / Pain / Weakness / Wound / Dental / dyspnoea / ear / eye / nose / throat / fever / boil / gastrointestinal / Sexually Transmitted Infection / gynaecological / male genital / urinary tract infection / Chest pain / musculoskeletal / preventive / skin and soft tissue / surgery-related / HIV related opportunist infections / other (specify) / unknown.
2. Today is [*select one option*] the expiry date. before / after / unknown
3. Did you receive written instructions for use? yes / no
4. Did you receive verbal instructions for use? yes / no
5. How do you rate your overall experience with the medicine supplier attended?

Visual analogue scale

We have finished the interview. Thank you for your participation.

**Table S1. Frequency of supplying watch-group antibiotics for selected populations**

| **ATC group** | **Watch antibiotic** | **Children**  **(n=389)** | **Private pharmacy**  **(n=863)** | **Non-prescription supply**  **(n=773)** | **Dental problem**  **(n=37)** | **Urinary tract problem**  **(n=36)** |
| --- | --- | --- | --- | --- | --- | --- |
| 2^nd^ Cephalosporins | Cefuroxime | 61 | 58 | 51 | 0 | 0 |
| 3^rd^ Cephalosporins | Cefixime | 64 | 70 | 48 | 1 | 0 |
|  | Cefdinir | 21 | 20 | 17 | 0 | 0 |
|  | Cefpodoxime | 10 | 39 | 28 | 0 | 0 |
| Macrolides | Azithromycin | 47 | 79 | 59 | 0 | 0 |
|  | Clarithromycin | 1 | 15 | 10 | 1 | 0 |
|  | Clindamycin | 0 | 4 | 1 | 1 | 0 |
|  | Erythromycin | 9 | 33 | 31 | 1 | 0 |
|  | Lincomycin | 2 | 27 | 18 | 5 | 0 |
|  | Spiramycin | 5 | 10 | 6 | 1 | 0 |
|  | Spiramycin & Metronidazole | 1 | 18 | 15 | 15 | 1 |
| Fluoroquinolones | Ciprofloxacin | 0 | 14 | 17 | 0 | 14 |
|  | Levofloxacin | 0 | 8 | 6 | 0 | 2 |
|  | Ofloxacin | 0 | 5 | 5 | 0 | 5 |

**Table S2. Accomplishment rate of number of encounters of 20 suppliers in the study sample**

| **Supplier no.** | **Type** | **Real number of encounters** | **Expected number of encounters** | **Accomplishment rate (=real/expected)** |
| --- | --- | --- | --- | --- |
| 1 | District hospital pharmacy | 109 | 120 | 90.8% |
| 2 | Polyclinical pharmacy | 114 | 120 | 95.0% |
| 3 | Private pharmacy | 92 | 120 | 76.7% |
| 4 | Private pharmacy | 73 | 120 | 60.8% |
| 5 | Commune health station | 54 | 120 | 45.0% |
| 6 | Private pharmacy | 60 | 120 | 50.0% |
| 7 | Commune health station | 67 | 120 | 55.8% |
| 8 | Private pharmacy | 57 | 120 | 47.5% |
| 9 | Private pharmacy | 75 | 120 | 62.5% |
| 10 | Private pharmacy | 60 | 120 | 50.0% |
| 11 | Private pharmacy | 44 | 120 | 36.7% |
| 12 | Private pharmacy | 57 | 120 | 47.5% |
| 13 | Private pharmacy | 52 | 120 | 43.3% |
| 14 | Private pharmacy | 47 | 120 | 39.2% |
| 15 | Private pharmacy | 65 | 120 | 54.2% |
| 16 | Private pharmacy | 57 | 120 | 47.5% |
| 17 | Commune health station | 89 | 120 | 74.2% |
| 18 | Private pharmacy | 74 | 120 | 61.7% |
| 19 | Private pharmacy | 64 | 120 | 53.3% |
| 20 | Private pharmacy | 94 | 120 | 78.3% |
